# Metagenomic Characterization of Bacterial Communities on Ready-to-Eat Vegetables and Effects of Household Washing on their Diversity and Composition

**DOI:** 10.3390/pathogens8010037

**Published:** 2019-03-19

**Authors:** Soultana Tatsika, Katerina Karamanoli, Hera Karayanni, Savvas Genitsaris

**Affiliations:** 1 School of Economics, Business Administration and Legal Studies, International Hellenic University, 57001 Thermi, Greece; s.tatsika@ihu.edu.gr; 2 Hellenic Food Safety Authority (EFET), 57001 Pylaia, Greece; 3 School of Agriculture, Aristotle University of Thessaloniki, 54124 Thessaloniki, Greece; katkar@agro.auth.gr; 4 Department of Biological Applications and Technology, University of Ioannina, 45100 Ioannina, Greece; hkaray@uoi.gr

**Keywords:** Ready-to-eat salads, 16S rRNA gene, illumina, household treatments, fresh produce, foodborne pathogens, *Pseudomonas*

## Abstract

Ready-to-eat (RTE) leafy salad vegetables are considered foods that can be consumed immediately at the point of sale without further treatment. The aim of the study was to investigate the bacterial community composition of RTE salads at the point of consumption and the changes in bacterial diversity and composition associated with different household washing treatments. The bacterial microbiomes of rocket and spinach leaves were examined by means of 16S rRNA gene high-throughput sequencing. Overall, 886 Operational Taxonomic Units (OTUs) were detected in the salads’ leaves. Proteobacteria was the most diverse high-level taxonomic group followed by Bacteroidetes and Firmicutes. Although they were processed at the same production facilities, rocket showed different bacterial community composition than spinach salads, mainly attributed to the different contributions of Proteobacteria and Bacteroidetes to the total OTU number. The tested household decontamination treatments proved inefficient in changing the bacterial community composition in both RTE salads. Furthermore, storage duration of the salads at refrigeration temperatures affected the microbiome, by decreasing the bacterial richness and promoting the dominance of psychrotropic bacteria. Finally, both salads were found to be a reservoir of opportunistic human pathogens, while washing methods usually applied at home proved to be inefficient in their removal.

## 1. Introduction

Plant associated bacteria have gained attention in recent years due to the potential relationships to human health in terms of the spread of foodborne pathogens and the contribution of edible plant diversity to human gut microbiomes [1,2]. Special attention has been given to the ecology of enteric human pathogens associated with fresh produce, as customer demands for fresh salads are continuously increasing and the presence of potentially pathogenic bacteria within plant-associated microbiota can affect produce safety [3,4,5]. Ready-to-eat (RTE) leafy vegetables are minimally processed products considered as foods that can be consumed immediately at the point of sale without further preparation or treatment. They are colonized by a variety of bacteria and recent outbreaks of human disease associated with fresh products have shown their vulnerability to colonization by foodborne pathogens such as *Escherichia coli* O157:H7 and *Salmonella enterica* [6,7]. European Food Safety Authority (EFSA) and European legislation placed leafy green vegetables (in particular lettuce and spinach) in the highest priority group for their strong involvement in disease outbreaks on a worldwide level [8]. European legislation is posing several microbiological criteria as indexes of the hygienic process and safety, proposing that the recovery of *E. coli* in RTE vegetables is an index of the hygienic process under which they are produced, and the recovery of *Salmonella* spp. and *Listeria monocytogenes* is an index of safety. 

Until recently, characterization of microbial communities of fresh cut and minimally processed vegetables (e.g., spinach) has focused on cultivation or microarray-based detection of bacteria associated to spoilage, and especially bacteria belonging to Pseudomonadaceae and Enterobacteriaceae [9,10]. Although the above-mentioned bacteria are frequently isolated from the phyllosphere of leafy vegetables, other bacteria present in lower numbers or non-cultivable bacteria could influence the quality, safety, and self-life of edible leaves due to the complex interactions among them on the vegetable leaves [11,12,13,14]. In recent years, the rapid development of Next Generation Sequencing (NGS) technologies have promoted the broad-spectrum identification of the whole microbiome in RTE salad leaves, without the need of the microbe cultivation step [15,16]. Recent studies using the 16S rRNA gene amplicon high-throughput sequencing (HTS) have suggested how salad leaf microbiome can be affected by season, irrigation, soil type and other parameters [17,18]. However, few studies investigating the leafy salad microbiomes have been focused on the final product at the point of consumption, such as bagged RTE salads subject to refrigerated storage [19]. The most common response pointed out in refrigerated food products was a reduction in bacterial diversity of the salad leaves (e.g., [20]). Also, the 16S rRNA gene HTS approach used to examine the composition of microbial communities on fresh spinach at two storage temperatures, 4 and 10 °C, allowed a broader description of the bacterial composition and diversity of the spinach salad than previously obtained using culture-based approaches, and suggested that HTS is a promising tool for safety and quality assurance of fresh cut vegetables [21]. Amplicon sequencing showed that the majority of leaf-associated bacteria in RTE salads are members of the Proteobacteria and Bacteroidetes groups [22], while at the same time, the method allowed the identification of numerous low abundance bacteria that could not be identified by culture-dependent methods [22]. Finally, Söderqvist et al. [23], using Illumina 16S rRNA gene amplicon sequencing showed that the composition of bacterial communities changed during cold storage (8 °C) of RTE baby spinach and mixed-ingredient salad, with Pseudomonadales (mostly represented by the genus *Pseudomonas*) being the most abundant high-level taxonomic group across the samples. Although there is a surge in studies using HTS to examine RTE salad microbiomes, to the best of our knowledge there are no studies that attempt to address whether the consumers can influence the bacterial composition and diversity of the RTE salads by household washing with clean water or with vinegar solutions. Even so, only few studies have examined the efficacy of household decontamination methods with culture-based methods (e.g., [24,25]). 

The aim of the current study was to determine the bacterial community composition of two different RTE leafy salads at the point of consumption and to investigate the changes in bacterial diversity and composition associated with different household washing treatments and different storage periods, using 16S rRNA gene HTS methods. Furthermore, the use of 16S rRNA gene HTS technologies can constitute a useful tool in the identification of potential taxa responsible for the spoilage of RTE salads.

In particular, the following hypotheses were made: the microbial composition of RTE leafy salads depends on both the type of the salad and on the storage period and refrigeration conditions; and, household washing methods are inefficient in the removal of potential opportunistic human pathogens.

## 2. Results and Discussion

In this study, high-throughput 16S rRNA gene amplicon sequencing was used to characterize the bacterial community composition associated with RTE leafy salads at the point of consumption. Specifically, the bacterial communities of RTE rocket salads were compared with the bacterial composition of RTE spinach salads. Furthermore, the community composition changes after different household treatments of the salads were also examined. Finally, differences in bacterial diversity and composition associated with different refrigerated storage duration of the rocket salad were considered. The samples coding according to type of salad, expiration date and treatment implemented is shown in Table 1.

Overall, 804,073 reads were recovered across the 24 samples, after downstream processing. The number of reads recovered per sample was not evenly distributed, ranging from 62,532 reads in RV1, to 18,590 in SW1. After normalization of the dataset to the lowest number of reads (i.e., 18,590 reads per sample), a total of 895 distinct OTUs were detected across all samples. Out of these 895 OTUs, 886 were found to closely relate to the domain Bacteria, while the remaining nine OTUs were closely affiliated to Eukaryota (Streptophyta), and thus removed from further analysis. Rarefaction curves calculated for all samples, approached a plateau in all cases when ≥ 97 % levels of sequence similarities were applied (Supplementary Figure S1). The ratio of observed to expected (*S*_chao1_) OTUs in the samples was 0.86 (mean ± 0.09) for all samples. Amongst the samples without any treatment, the lowest Simpson, Shannon and Equitability indexes were recorded in the two rocket salads with the expiring date of 20-06-2018 (RN1: 0.62, 1.80, 0.37 and RN2: 0.73, 2.00, 0.41 respectively) while the highest values were calculated for spinach salads (SN2: 0.89, 2.85, 0.56, SN3: 0.88, 2.72, 0.52 and SN4: 0.90, 2.96, 0.58 respectively) (Supplementary Table S1). The storage at refrigeration temperatures appeared to affect the bacterial composition of rocket salad, decreasing overtime the richness, the diversity and the evenness of the microbiome.

### 2.1. Bacterial Diversity and Composition of RTE Salads

According to Kolmogorov-Smirnov test for equal distributions, no significant differences were detected between the bacterial taxonomic profiles of salad replicates (i.e., the subsamples taken from different packages of the same salad) by pairwise comparisons of either bacterial abundances or α-diversity estimators (p > 0.05 in all pairwise comparisons). This way the high similarity of the bacterial taxonomic profiles between the four product packages of each salad was verified, and therefore in following result presentations, the average OTUs richness/abundances between the four replicates will be used, when applicable.

**Figure 1:**
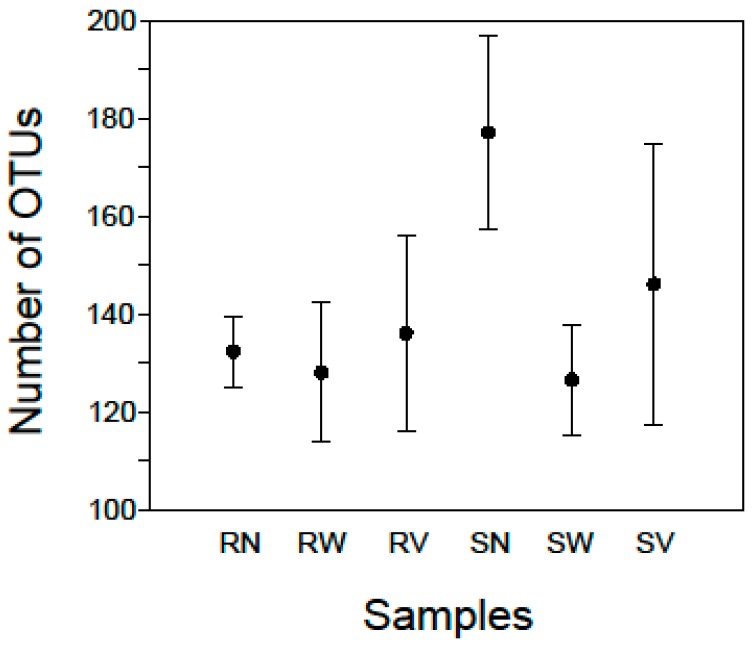
Mean numbers of OTUs in rocket (R) and spinach (S) salad samples with no treatment (N) and after water (W) and vinegar (V) solution treatments. Standard error bars are shown.

At the lowest taxonomic resolution, between 113 and 199 OTUs (141 mean ± 24 SD) were detected in each sample (Figure 1; Supplementary Table S1). For each sample of rocket salad with no treatment a mean of 132.25 (±7.2 SD) OTUs was detected while the corresponding number for samples with spinach salad was a mean of 177 (±19.7 SD) OTUs (Figure 1), indicating that the bacterial community of RTE spinach salad was more diverse than that of rocket salad. The diversity found in our samples overall can be attributed to the fact that the vegetables had been minimally processed, packaged, transported and maintained under refrigeration conditions at the retail store before being analyzed. Lopez-Velasco et al. [20] using pyrosequencing of 16S rRNA genes identified more than 1000 OTUs in fresh spinach leaves, albeit using 99% similarity levels of OTUs clustering, making direct comparisons with our samples biased. Jackson et al. [22] also using 16S pyrosequencing detected 22 and 57 OTUs in RTE baby spinach samples in conventional and organic varieties respectively. Leff and Fierer [26] reported slightly higher OTU numbers for surface bacterial communities on store-bought spinach (approximately 50 OTUs for conventional and 65 OTUs for organic varieties). These studies can give an approximation of the expected bacterial diversity in spinach salads, showing a relatively rich bacterial community, but to the best of our knowledge no study has been published on RTE rocket salad bacterial diversity by means of high throughput sequencing.

**Figure 2:**
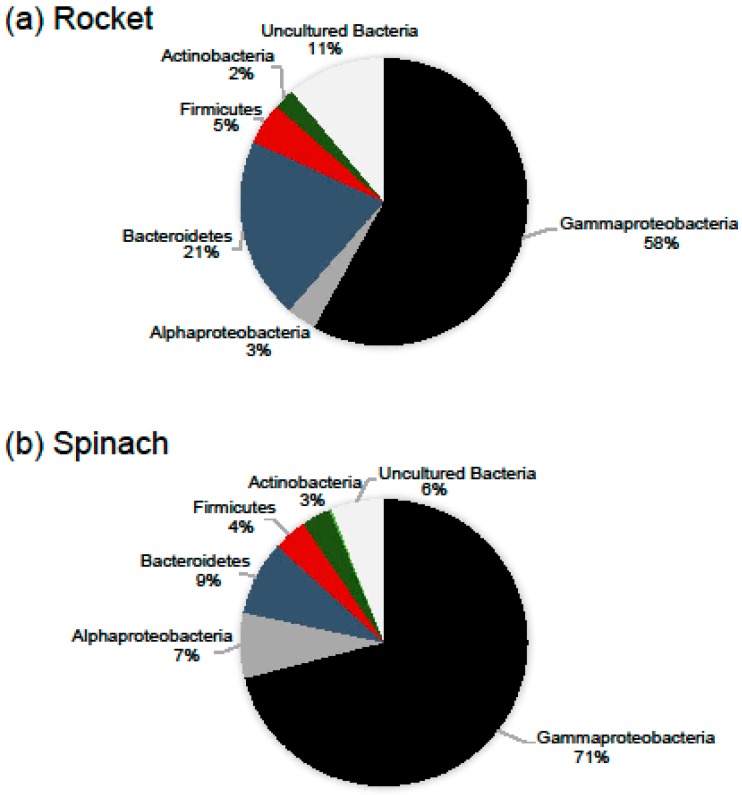
Relative number of OTUs belonging to major high-level bacterial taxonomic groups, based on the data from all samples. Taxonomic assignment of OTUs was based on SINA searches against the SILVA 132 database, after verification searches against GenBank.

Besides OTUs richness, the leaves of the two types of salads showed different bacterial composition. In particular, Proteobacteria was the dominant phylum in the leaves of the two RTE salads (comprising of 67.4% of the total number of OTUs) with Gammaproteobacteria being the most diverse subphylum, followed by Bacteroidetes (13.3%), Firmicutes (5.1%) and Actinobacteria (2.3%). While Gammaproteobacteria was the richest subphylum in all samples, the portion of other phyla and subphyla varied. The most notable difference between the two salad types was found to be on the diversity of Proteobacteria and Bacteroidetes. Proteobacteria and Bacteroidetes contribution to the total number of rocket salad OTUs was found to be 61.4% and 20.5% and to the total number of spinach salad OTUs 78.3% and 8.4 %, respectively (Figure 2). A percentage of 82.7% of the reads obtained from the leaves of rocket salad were annotated to Proteobacteria-related OTUs, followed by Bacteroidetes-related reads (up to 16.3% of the reads). On the other hand, 94.1% and 3.7% of the reads obtained from the leaves of spinach salad belonged to Proteobacteria and Bacteroidetes-related OTUs, respectively. The bacterial taxonomic groups detected in our samples were consistent with findings from other studies that have used culture independent techniques for the identification of surface bacterial communities of leafy salads [20,26,27,28]. Jackson et al. [22] identified Gammaproteobacteria and Betaproteobacteria as the dominant lineages in almost all RTE leafy salads examined, which accounted for at least 90% of the reads obtained. 

**Figure 3:**
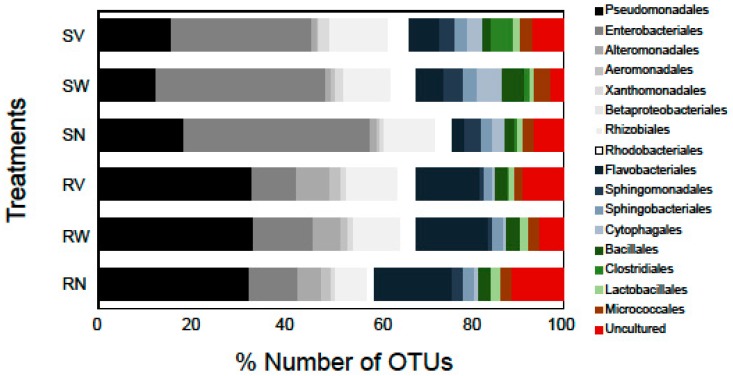
Number of OTUs (%) of high-level bacterial taxonomic groups (order level) detected in RTE salad samples. The labels SN, SW, SV represent samples from RTE spinach salad without any treatment and with water and vinegar treatment, respectively. The labels RN, RW, RV represent samples from RTE rocket salad without any treatment and with water and vinegar treatment, respectively. Shades of grey represent Proteobacteria, shades of blue represent Bacteroidetes, and shades of green represent Firmicutes. Bacterial orders with OTU richness < 1% in all data sets are not shown.

Bacterial taxa such as Enterobacteriales and Pseudomonadales, commonly isolated using culture-based and culture-independent studies (e.g., [20,29]), were also identified in our study in high relative abundances suggesting that these families could be among the characteristic dominant families of minimally processed leafy salads. The most dominant bacterial order in terms of OTUs number in rocket salad samples was found to be Pseudomonadales (32.2% of the total number of OTUs), while in spinach salad it was the order Enterobacteriales (39%). Flavobacteriales was the second most dominant order in rocket salad (16.4%) and Enterobacteriales was the third (10.4%). In spinach samples, the second most dominant order was Pseudomonadales (17.9%) followed by Betaproteobacteriales (10.6%) (Figure 3). The relative number of OTUs of Rhizobiales and Cytophagales were markedly higher in all spinach samples in comparison with rocket samples (Figure 3). On the other hand, the Alteromonadales had a higher relative number of OTUs in all rocket samples comparing to spinach samples. Notable differences were also detected in the relative abundances of these bacterial taxa between different RTE salads (data not shown). 

Members of Enterobacteriales order are usually found in human and other animals’ gut, while others are found in water and soil or as plant pathogens. Members of the Pseudomonadales order mostly represented by the *Pseudomonas* genus have widespread occurrence in water and plant seeds [30,31]. Thus, these bacteria can originate both from the raw vegetables and from the post-harvesting handling environment (as a result of cross contamination). Our results showing distinct differences in the bacterial diversity, composition and taxa relative abundances between the two types of salads, suggest that the microbiota of RTE leafy salads at the time of purchase and consumption is indicative primarily of the microbiota present on the respective growing plant. Consumers, thus, seem to be exposed to substantially different microbiota, depending on the type of the RTE salad they consume. 

### 2.2. Bacterial Diversity and Composition after Household Treatments

For the rocket salad samples after water treatment (RW) a mean of 128 (± 14.3 SD) OTUs was detected while the corresponding number for spinach salad samples (SW) was 126.5 (± 11.2 SD) OTUs (Figure 1). For the rocket samples after vinegar solution treatment (SV) a mean of 136 (± 19.9 SD) OTUs was detected while the corresponding number for spinach samples was 146 (± 28.6 SD) OTUs (Figure 1). This indicates that for RTE rocket salad no remarkable changes were observed after household treatments but there was a reduction in the richness of bacterial communities of RTE spinach salad. On the other hand, the samples with the highest Simpson Index scores came from rocket salad after water treatment (RW4) 0.91 and spinach salad after vinegar treatment (SV3) 0.92 (Supplementary Table S1), indicating that household treatments did not reduce the diversity of the microbial communities in RTE salads. 

Our results also showed that a substantial part of the RTE spinach salad microbiome after household treatments still consisted of Enterobacteriales-related OTUs (e.g., the genera *Enterobacter*, *Serratia*, *Pantoea*), Pseudomonadales and Burkholderiales (e.g., genus *Janthinobacterium*) and that a substantial part of rocket salad microbiome after household treatments still consisted of Pseudomonadales (e.g., genera *Pseudomonas*, *Acinetobacter*) and Flavobacteriales (e.g., genus *Flavobacterium*), which include potentially pathogenic members. These results indicate that possible consumers cannot influence the bacterial composition and diversity of RTE vegetables by implementing the common household washing treatments applied in the present study. 

**Figure 4:**
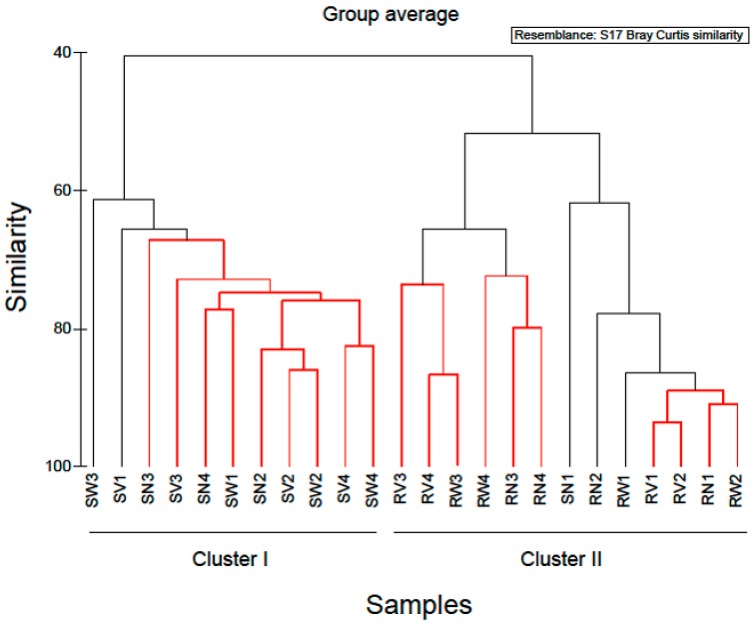
Cluster diagram based on Bray-Curtis dissimilarities calculated based on the non-transformed number of reads of OTUs found during the study. Red clades in the dendrogram indicate significant bifurcations, based on the SIMPROF significance test. The labels SN, SW, SV represent samples from RTE spinach salad without any treatment and with water and vinegar treatment, respectively. The labels RN, RW, RV represent samples from RTE rocket salad without any treatment and with water and vinegar treatment, respectively.

In order to examine the variations in community structure between all vegetable samples, the Bray-Curtis dissimilarity index was used, and visualized through a dendrogram (Figure 4). Hierarchical clustering grouped RTE vegetable samples into two distinct groups. The first cluster comprised of almost all spinach salad samples and the second cluster comprised of all rocket salad samples. Based on this clustering, it appeared that spinach salad samples have different bacterial community structure than rocket salad samples and most notably, this was independent of the household treatments. Similarly, Uhlig et al. [25] using culture-dependent and independent methods, showed the inefficiency of tap water washing methods available at home to remove bacteria from lettuce below safe limits, demonstrating the responsibility of the producers and distributors to ensure the hygienic quality of the green produce. Furthermore, experiments investigating the effect of exposure to acetic acid solution (0.5% and 1.0% v/v) at reducing *Listeria monocytogenes* numbers on salad vegetables, indicated that the efficacy of this method was limited and varied with vegetable type [24]. Thus, if bacteria with pathogenic potential are present in these RTE vegetables, they are likely to resist removal even by further washing. Moreover, there is always the risk of cross-contamination from handlers and food contact surfaces in case of improper handling at home or mass catering premises during additional washing.

Simper analysis identified OTUs which contributed most to the dissimilarity of the bacterial communities between rocket and spinach samples (Table 2). Twenty-eight OTUs contributed > 90% to the dissimilarity of the bacterial communities between rocket and spinach samples (see Table 2). The majority of them (22 out of 28) belonged to Gammaproteobacteria, while the remaining were affiliated to Bacteroidetes (3/28), Firmicutes (2/28) and Alphaproteobacteria (1/28) (Table 2). Among the closest relatives of these OTUs, clones retrieved from plant rhizospheres, other commercial vegetables and soil environments were detected. Also, the species like *Acinetobacter johnsonii*, (OTU002), *Pseudomonas aeruginosa* (OTU005), *Pantoea agglomerans* (OTU0011) and *Enterobacter* sp. (OTU0015) are considered as opportunistic pathogens with the potential to generate antibiotic resistance or multi resistance.

Based on the hierarchical clustering (Figure 4) used to examine the variations in community structure between all vegetable samples, one spinach sample (SN1) was clustered with rocket salad samples with the expiring date of 20-06-2018. Simper analysis identified OTUs which contributed most to the dissimilarity of the bacterial communities between SN1 and SN2–SN4 (no treatment). Comparing the relative abundances of the bacterial composition at family level and at genus level, in all RTE spinach samples with no treatment, it was clear that in SN1 the dominant bacteria family was Pseudomonadaceae (represented by the genus *Pseudomonas*) and in the rest of the samples the dominant bacteria family was Enterobacteriaceae (mostly represented by the genera *Erwinia, Pantoea, Serratia and Enterobacter*). Due to the fact that all the bags of RTE spinach salad were from the same production batch, a possible explanation of the above results, is that a part of the raw spinach used for the production of the RTE salads was stored under refrigeration conditions for a sufficient period of time before processing, giving the opportunity to psychrotrophs like *Pseudomonas* spp. to dominate among the bacterial populations. Taking this into account, it is suggested that the microbiome present in and on RTE leafy vegetables at the time of purchase and consumption also depends on post-harvest handling conditions, especially storage temperature [19]. 

### 2.3. Changes in RTE Salad Microbiome during Refrigerated Storage

**Figure 5:**
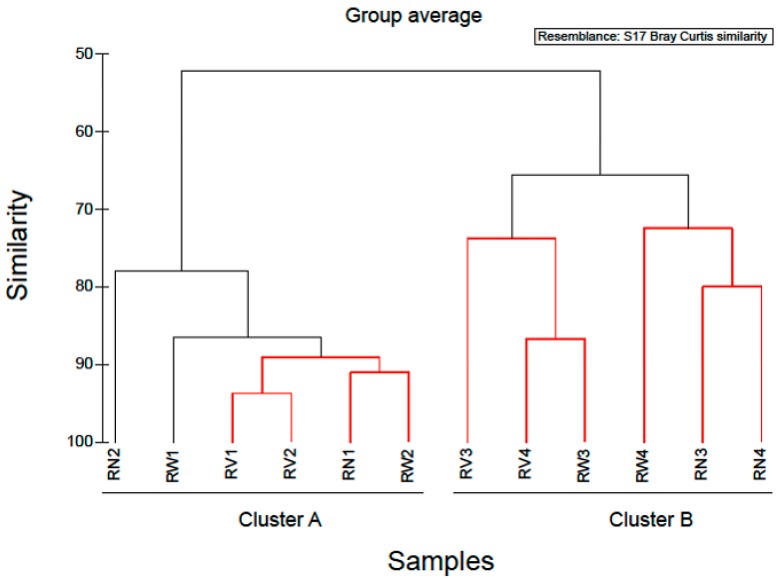
Cluster diagram based on Bray-Curtis dissimilarities calculated based on the non-transformed number of reads of OTUs found in RTE rocket salad samples. Red clades in the dendrogram indicate significant bifurcations, based on the SIMPROF significance test. The labels RN, RW, RV represent samples from RTE rocket salad without any treatment and with water and vinegar treatment, respectively. Numbers 1 & 2 represent packages with expiring date of 23 June 2018, whereas numbers 3 & 4 represent packages with expiring date of 20 June 2018.

Hierarchical clustering separated the rocket salad samples into two distinct groups (Figure 5). The first cluster comprised of all rocket salad samples with the expiring date of 20 June 2018 and the second, all rocket salads with the expiring date of 23 June 2018. A notable increase in the relative abundance of Pseudomonadales (mostly represented by the genus *Pseudomonas*) and a reduction in relative abundance of Enterobacteriales was found in rocket salad with expiring date of 20 June 2018, comparing to rocket salad with expiring date of 23 June 2018. The clustering of the rocket salads in two groups might simply reflect the dissimilarity between two different sampling batches. However, it might also be an indication that storage of RTE salads at refrigeration temperatures could affect the bacterial composition in higher taxonomic levels, decreasing overtime the abundance of Enterobacteriales and increasing the abundance of Pseudomonadales (Pseudomonadaceae and Moraxellaceae family). In order to produce more definite conclusions on the effects of refrigeration duration on the bacterial composition of RTE salad leaves, a time-course test by sampling at different storage times and more biological replicates are necessary. Our results present an initial indicator towards future research on RTE salad refrigeration storage duration and subsequent spoilage, by attempting the characterization of bacterial taxa that could contribute to salad spoilage. 

Simper analysis identified OTUs that contributed most to the dissimilarity of the bacterial communities between rocket salads of different production dates (data not shown). Nineteen OTUs contributed > 90% to the dissimilarity of bacterial communities between rocket salads. The majority of them (14 out of 19) belonged to Gammaproteobacteria, while the rest were affiliated to Bacteroidetes (4/19) and Firmicutes (1/19). Bacteria capable of surviving under refrigeration conditions, known as psychrotrophs, have been linked to the spoilage of refrigerated foods like leafy vegetables [30]. Members of the Pseudomonadales like *Pseudomonas* spp. are known psychrotrophs and were found to be among the dominant bacterial populations in RTE spinach and mixed vegetable salads during cold storage [20,23,27]. They are widely distributed in the nature with some species recognised as potential plant pathogens and others as playing an important role in spoilage of green leafy vegetables due to their ability to produce pectinolytic enzymes that can cause soft rot of fleshy vegetables [30]. 

### 2.4. Potential Pathogens and Other Notable OTUs

The High Throughput 16S rRNA gene Sequencing is recognized as a powerful tool to reveal previously undetected or overlooked bacterial diversity (e.g., [32]). However, it can also comprise of limitations concerning strain identification and subsequent possible functional distinctions among bacteria within the same genera, based on the produced read length (e.g., discussed in [33]). In the present study, the produced read length was approximately 470 bp, and phylogenetic resolution was limited in order to link OTUs to specific species and functions with certainty. The results presented in the following paragraphs are based on the closest relatives of dominant and other notable OTUs, with noteworthy characteristics concerning the consumption of RTE salads according to the literature.

Across all samples, the five most abundant OTUs were found to be closely related to clones of Pseudomonas frederiksbergensis, Acinetobacter johnsonii, Erwinia rhapontici, Rheinheimera sp., and Pseudomonas aeruginosa (Table 2). Pseudomonas frederiksbergensis is a Gram-negative, phenanthrene-degrading bacterium [34]. The presence of this OTU in high relative abundances, especially to rocket salad samples close to their expiring date, could suggest that this bacterium may play an important role in product degradation. *Acinetobacter johnsonii* is a gram – negative bacterium usually found in the environment and animals; it can occasionally colonize human skin and rarely causes nosocomial infections [35]. Its importance in RTE salads is unknown. Erwinia rhapontici is also a gram – negative bacterium, a known opportunistic plant pathogen that attacks a wide range of plant hosts causing pink seed and crown rot or soft rot [36]. Other known genera of soft-rot bacteria are Xanthomonas, Pseudomonas, Clostridium and Bacillus. While soft-rot Erwinia can be active only at temperatures above 20 °C, the fluorescent Pseudomonas (Pseudomonas fluorescens, Pseudomonas viridiflava) can decay plant tissue at temperatures below 4 °C [37]. In our samples, OTU009 was found to be closely related to Pseudomonas viridiflava, a bacterium with high prevalence on decayed vegetables at wholesale and retail markets [38]. Rheinheimera have been isolated from soil in South Korea and from irrigation water [39,40] and its role in RTE salads is unknown (no relative publications were found). Pseudomonas aeruginosa is a known plant pathogen found in soil, water, skin flora, and most man-made environments throughout the world. It can become virulent in those immunocompromised, or people with an underlying disease [41].

Generally, the vegetable microbiome is recognised as a reservoir of several opportunistic pathogens [1]. Once ingested with foods, these microorganisms may survive in the gastrointestinal track and spread throughout the gut, where they can cause infections [42]. Except from *Pseudomonas aeruginosa*, in our study further dominant OTUs were found to be closely affiliated to some other genera that contain species which are potential human pathogens, e.g., the genera *Pantoea*, *Serratia*, *Kluyvera*, and *Enterobacter.* These genera were found in relative abundances above 1% in spinach samples and in lower relative abundances (< 1%) in rocket salad samples, before and after household washing treatments. One OTU (OTU028), detected in all samples, was found to be closely related to *Stenotrophomonas maltophilia*, an environmental global emerging multidrug resistant nosocomial pathogen, ubiquitous in aqueous environments, soil, and plants, with the ability to form biofilms [43]. Previous studies have suggested that antibiotic resistant bacteria can be present on fresh produce, which means that vegetables can act as a source for the spread of antibiotic resistance, regardless of whether or not the bacteria are able to grow on that matrix [44,45,46]. The current results show the inefficiency of the tested household washing treatments to remove such bacteria from RTE salads. 

Apart from potential human pathogens, a number of OTUs closely affiliated to taxa of animal and plant pathogens, were also recovered. For example, OTU018, closely related to *Aeromonas hydrophyla*, was found in almost all of the vegetable samples in relative abundances above 1% and is considered a pathogen of fish and amphibians but also it has been implicated in diarrheal disease in humans [47]. *A. hydrophyla* has been found to form a biofilm in the leaves of green vegetables and this must be taken into account when washing them before consumption [48]. Furthermore, OTU033, closely related to *Shewanella putrefaciens*, was found in relative abundances below 1% in almost all of the salad samples before and after washing is associated with fish spoilage [49]. However, it was previously isolated from hydroponic lettuce cultivation systems and RTE salads [25,50]. Based on the literature, *Shewanella* is known to cause problems by creating biofilms on food processing surfaces and its presence may indicate marine source of irrigation water or contaminated processing surfaces [51]. Although it is very rare to act as a human pathogen, cases of infections and bacteraemia have been reported [52].

European legislation on food safety, is posing several microbiological criteria through the EC Regulation no. 1441/2007 (that amended the EC regulation no. 2073/2005) [53], indicating the recovery of *E. coli* in RTE vegetables as an index of the hygienic process under which they are produced (is considered as an indicator of faecal contamination) and *Salmonella* spp. and *Listeria monocytogenes* as an index of safety. None of these taxa, or other foodborne pathogenic taxa, were found in the present study.

## 3. Materials and Methods

### 3.1. Sample Processing

In total, eight packages of commercial ready-to-eat (RTE) leaf salads were randomly purchased from a supermarket in Thessaloniki, Greece, on 19 June 2018. The bagged vegetables were Private Label products, produced at the same production facilities (by a Greek food industry as it was stated on the labeling of the products). Four of them consisted of intact rocket (ruckola) leaves and the other consisted of sliced (chopped) leaves of spinach and were stored in the chilled produce section (storage temperature during sampling: 4.9 °C). The salads were packaged as leaves or leaf pieces, with just a single type of vegetable per pack and were labeled as “ready to eat”. The expiring date for two rocket salad packages (selected by chance) were 20/06/2018 (likely date of production 13 June 2018; RN1 & RN2) and for the other two rocket packages (also selected by chance) the expiring date was 23/06/2018 (likely date of production 16 June 2018; RN3 & RN4). Thus, for the RTE rocket salad, two different production batches have been selected (two packages from each batch); with the likely date of production to differ between batches (three-day interval). The bags with RTE spinach were all from the same production batch. For all the packages of spinach, also selected by chance, the expiring date was 20 June 2018 (likely date of production 16 June 2018) (Table 1). Samples were collected one hour prior to laboratory procedures and stored in a portable isothermal refrigerator (4 °C) prior to processing. 

In the laboratory, subsamples of 0.5–1 g of leaves were collected as follows: (a) Eight subsamples were collected directly from each package, meaning four subsamples from the four rocket packages (RN1–RN4) and four from the spinach packages (SN1–SN4). (b) Four rocket subsamples were collected after dipping and stirring vigorously 50 g of rocket leaves in a bowl of tab water for 1 min (RW1–RW4) and four subsamples were similarly obtained after following the same procedure for spinach (SW1–SW4). (c) Four rocket subsamples were collected after dipping and stirring 50 g of rocket leaves in 1% concentration vinegar solution for 1 min (RV1–RV4) and four subsamples of spinach leaves were also obtained after following the same procedure (SV1–SV4) (Table 1).

The samples were placed in test tubes with 10 ml PBS buffer (NaCl 137 nmol L^−1^, KH_2_PO_4_ 1.8 nmol L^−1^, KCl 2.7 nmol L^−1^ and Na_2_HPO_4_ 1.42 nmol L^−1^, pH = 7.4) and sonicated for 10 min (Transsonic 460). The solutions (without the leaves) were subsequently centrifuged at 9500 rpm for 20 min, and the sedimentation material was placed in −20 °C, until further processing. The supernatant fluid from each tube was discarded. Thus, a total of 24 subsamples were collected, 12 from the spinach salad and 12 from the rocket salad, i.e., four subsamples for each treatment per salad type. 

### 3.2. DNA Extraction and Sequencing

DNA was extracted from the 24 subsamples using a Macherey–Nagel NucleoSpin^®^ Soil, Genomic DNA Isolation Kit, according to the manufacturer’s instructions. The concentration and quality of recovered DNA was confirmed using the Thermo Scientific™ NanoDrop™ spectrophotometer.

The extracted DNA was subjected to PCR using specific primers targeting the V6-V8 hyper variable region of the 16S rRNA gene (B969F = ACGCGHNRAACCTTACC, BA1406R = ACGGGCRGTGWGTRCAA). These primers have been found to successfully amplify approximately 470 bp in the V6-V8 hypervariable region of all the major high-level bacterial taxonomic groups [54]. Next, the PCR products were purified and amplicon samples were sequenced on Illumina MiSeq using 300+300 bp paired-end chemistry which allows for overlap and stitching together of paired amplicon reads into one full-length read of higher quality (http://cgeb-imr.ca/protocols.html). The Illumina Nextera Flex kit for MiSeq+NextSeq, which requires a very small amount of starting material (1 ng), was used. The PCR amplification step and the sequencing were performed at the Integrated Microbiome Resource (IMR) at Dalhousie University, (Halifax, NS, Canada) 

### 3.3. Read Processing

The produced reads were subjected to downstream processing using the *mothur* v.1.34.0 software [55], following the proposed standard operating procedure [56]. Briefly, forward and reverse reads were joined, and contigs below 200 bp, with >8 bp homopolymers and ambiguous base calls were removed from further analysis. The remaining reads were dereplicated to the unique sequences and aligned independently against the SILVA 132 database, containing 1,861,569 bacterial SSU rRNA gene sequences [57]. Then, the reads suspected for being chimeras were removed using the UCHIME software [58]. The remaining reads were clustered into Operational Taxonomic Units (OTUs) at 97% similarity level. In order to obtain a rigorous dataset, OTUs with a single read in the entire dataset were removed from the analysis, as they were suspected of being erroneous sequences (e.g., see [59,60,61]). The resulting dataset was normalized to the lowest number of reads found in one sample (i.e., 18,590 reads), using the subsample command in *mothur*. The normalization process, although leading to small loss of the rare diversity, is considered essential to diversity estimate comparisons among different samples (e.g., [55]), and it was implemented as a good compromise in order to attain meaningful ecological comparisons between samples (e.g., see [62,63,64]). About 10% of the total number of OTUs were removed, all of them with relative abundances < 0.1% of the total number of reads. Taxonomic classification on the remaining OTUs was assigned using the SINA searches on the SILVA 132 curated database [65] and verified according to BLAST searches on GenBank. No chloroplast and mitochondria-related OTUs were recovered after downstream process and taxonomic annotations. The reads belonging to OTUs affiliated to unclassified sequences at the domain level were removed from the dataset in order to be confident that the produced dataset included only bacterial reads. Finally, eukaryotic reads were also removed. The raw reads were submitted to GenBank-SRA under the accession number PRJNA517014.

### 3.4. Data Analysis

The Kolmogorov – Smirnov test for equal distributions was used to assess significant differences in the relative abundance distributions of bacterial taxonomic profiles between replicates (SN1 to SN4 and RN1 to RN4), using the PAST v.3 software [66]. Furthermore, rarefaction curves, the richness estimator *S*_chao1_, and α-diversity estimators, i.e., the Shannon, Simpson and Equitability indexes, were also calculated with the PAST v.3 software in all 24 samples. The bacterial assemblages of the different samplings were compared using the Plymouth routines in the multivariate ecological research software package PRIMER v.6 [67]. The Bray-Curtis dissimilarity coefficients were calculated to construct the matrix based on OTUs abundances to identify interrelationships between samples and construct the cluster plots. The similarity profile (SIMPROF) permutation test was conducted in order to calculate the significance of the dendrogram branches resulting from cluster analysis. The similarity percentage analysis (SIMPER) was used for the specification of those OTUs which were responsible for the within group similarities and between group dissimilarities [68].

## 4. Conclusions

The results of our study show that microbial communities in RTE vegetable salads can be diverse and that microbial composition mainly depends both on the type of the raw material and on the storage conditions before and after processing. RTE salad microbiome comprised of OTUs closely related to a number of genera/higher taxa which include opportunistic human pathogens. The washing methods usually available at home proved to be inefficient in the removal of such taxa. More work is needed in order to assess if these bacteria represent a health risk in RTE vegetable salads, especially for immunocompromised people, taking into account the fact that all those bacteria are common inhabitants in the environment and in fresh vegetables. No foodborne pathogenic taxa were found in the present study with the use of High Throughput Sequencing of the 16S rRNA gene. The estimation of RTE salad microbiota with this technology can constitute a very useful tool in the characterization of the whole bacterial community and the identification of potentially pathogenic taxa to humans, and taxa responsible for the spoilage of the product. 

## Figures and Tables

**Table 1:** Samples of ready-to-eat (RTE) leafy salads, expiring date, treatment and coding used in the text.

RTE Salad	Expiring Date	Number of Replicates	Treatment	Code
Rocket	20 June 2018	2	No treatment	RN1–RN2
			Water	RW1–RW2
			Acetic Acid Solution	RV1–RV2
Rocket	23 June 2018	2	No treatment	RN3–RN4
			Water	RW3–RW4
			Acetic Acid Solution	RV3–RV4
Spinach	23 June 2018	4	No treatment	SN1–SN4
			Water	SW1–SW4
			Acetic Acid Solution	SV1–SV4

**Table 2:** SIMPER percentage contribution of typifying taxa to the dissimilarity of the bacterial communities between rocket (R) and spinach (S) salad samples according to Bray-Curtis dissimilarities (see Figure 4), their putative higher taxonomic affiliation, their closest relative based on BLAST searches against SINA and GenBank database, the isolation source of the closest relative, and their relative abundance on the total number of reads. Coding of salad samples is indicated in Table 1.

OTUs	Putative High-Level Taxonomic Affiliation	Closest Cultured Relative (% Similarity) [Accession Number]	Isolation Source	SIMPER Contribution (%)	SIMPER Cumulative Contribution (%)	RTE Salad	Relative Abundance (%)
OTU001	Gammaproteobacteria	*Pseudomonas frederiksbergensis* [MH144327]	Soil at a coal gasification site	20.51	20.51	All	35.2
OTU003	Gammaproteobacteria	*Erwinia rhapontici* [LC424328]	Barkey	10.78	31.28	All	7.4
OTU002	Gammaproteobacteria	*Acinetobacter johnsonii* [MK294307]	Mine tailings	8.77	40.05	All	7.9
OTU004	Gammaproteobacteria	*Rheinheimera* sp. [LC270228]	Wastewater stream	7.58	47.64	All rocket samples & SN2 SV1 SV3 SW1 SW2	4.8
OTU008	Gammaproteobacteria	*Serratia* sp. [KU750792]	Rhizosphere from Lepidium meyenii	5.08	52.72	All	3.7
OTU0010	Gammaproteobacteria	*Pantoea agglomerans* [MG681225]	Commercial Cucumber Fermentation Cover brine	4.51	57.23	All	2.8
OTU007	Bacteroidetes	*Flavobacterium resistens* [MH549189]	Insuyu cave	4.32	61.55	All	3.4
OTU0011	Gammaproteobacteria	*Pantoea agglomerans* [MH101508]	Chelidonium majus (medical herb)	4	65.56	All	2.5
OTU0014	Gammaproteobacteria	*Pantoea* sp. B1(2013) [KF010367]	Plant root	3	68.55	All spinach samples & RN3RN4RV3RV4RW2RW3RW4	1.8
OTU005	Gammaproteobacteria	*Pseudomonas aeruginosa* [MF838682]	Dairy product	2.72	71.27	All	4.3
OTU0015	Gammaproteobacteria	*Enterobacter* sp. [MG681230]	Commercial Cucumber Fermentation Cover brine	2.08	73.35	All	1.7
OTU0016	Gammaproteobacteria	*Kluyvera intermedia* [MH620740]	-	1.8	75.16	All	1.5
OTU009	Gammaproteobacteria	*Pseudomonas viridiflava* [MG972916]	Fresh-cut escarole	1.69	76.85	All	2.8
OTU0012	Gammaproteobacteria	*Janthinobacterium* sp. [MF774126]	Himalayan region	1.5	78.35	All	2.1
OTU0013	Gammaproteobacteria	*Duganella zoogloeoides* [KT983992]	Fresh water	1.49	79.84	All	2.1
OTU0017	Bacteroidetes	*Chryseobacterium indoltheticum* [MK138643]	Marine mud	1.31	81.14	All	1.2
OTU0019	Alphaproteobacteria	*Sphingomonas faeni* [MH482321]	Indoor dusts in animal sheds	1.28	82.42	All except RN1. RW1. RV1	0.8
OTU0018	Gammaproteobacteria	*Aeromonas hydrophila* [MK038972]	Freshwater cage culture system	1.03	83.46	All except SW2	0.9
OTU0021	Firmicutes	*Paenibacillus* sp. [MH769399]	-	0.98	84.44	All	0.7
OTU0023	Gammaproteobacteria	*Comamonas jiangduensis* [MH712950]	*“Oncorhynchus tshawytscha”* (Salmon)	0.94	85.38	All except SW2. SW4. SV2	0.7
OTU0026	Gammaproteobacteria	*Enterobacter ludwigii* [MH137696]	“Plutella xylostella” (diamondback moth)	0.83	86.21	All	0.5
OTU0031	Gammaproteobacteria	*Pantoea ananatis* [KX891513]	-	0.77	86.98	All spinach samples & RN2RN3RV3RV4RW3RW4	0.5
OTU0020	Gammaproteobacteria	*Massilia aurea* [KY047391]	Wetland. Drinking water distribution system	0.65	87.63	All	0.7
OTU0027	Gammaproteobacteria	*Pectobacterium zantedeschiae* [MG761827]	“Zantedeschia sp. (calla lily)” Tubers	0.61	88.24	All	0.6
OTU0032	Gammaproteobacteria	*Erwinia* sp. [MG859640]	Skin	0.57	88.81	All	0.4
OTU0030	Gammaproteobacteria	*Acidovorax* sp. [JQ723711]	Chelidonium majus root	0.52	89.33	All except SW4	0.4
OTU0024	Firmicutes	*Exiguobacterium antarcticum* [MH125158]	Soil	0.5	89.83	All	0.6
OTU0022	Bacteroidetes	*Sphingobacterium faecium* [MK100919]	Rhizosphere and roots of wheat	0.5	90.33	All	0.7

## Supplementary Materials

The following are available online at https://www.mdpi.com/2076-0817/8/1/37/s1, Figure S1. Rarefaction curves representing the number of OTUs against the number of high-quality reads, Table S1: OTUs number, the richness estimator Schao1 and α-diversity measurements (Simpson, Shannon and Equitability) per sample.
