# Supplementary material for: Metagenomic Characterization of Bacterial Communities on Ready-to-Eat Vegetables and Effects of Household Washing on their Diversity and Composition
Source: Pathogens. 2019 Mar 19;8(1):37. doi: 10.3390/pathogens8010037 (PMC6471099; doi:10.3390/pathogens8010037)
Supplement: Supplementary file 1 [file pathogens-08-00037-s001.pdf]

pathogens

MDPI

Article

Supplementary Material: Metagenomic Characterization of Bacterial Communities on Ready-to-Eat Vegetables and Effects of Household Washing on their Diversity and Composition

Soultana Tatsika [1,2], Katerina Karamanoli [3], Hera Karayanni [4] and Savvas Genitsaris [1,*]

1 School of Economics, Business Administration and Legal Studies, International Hellenic University, Thermi, 57001, Greece
2 Hellenic Food Safety Authority (EFET), Pylaia, 57001, Greece
3 School of Agriculture, Aristotle University of Thessaloniki, Thessaloniki, 54124, Greece
4 Department of Biological Applications and Technology, University of Ioannina, Ioannina, 45100, Greece
* Correspondence: s.genitsaris@ihu.edu.gr

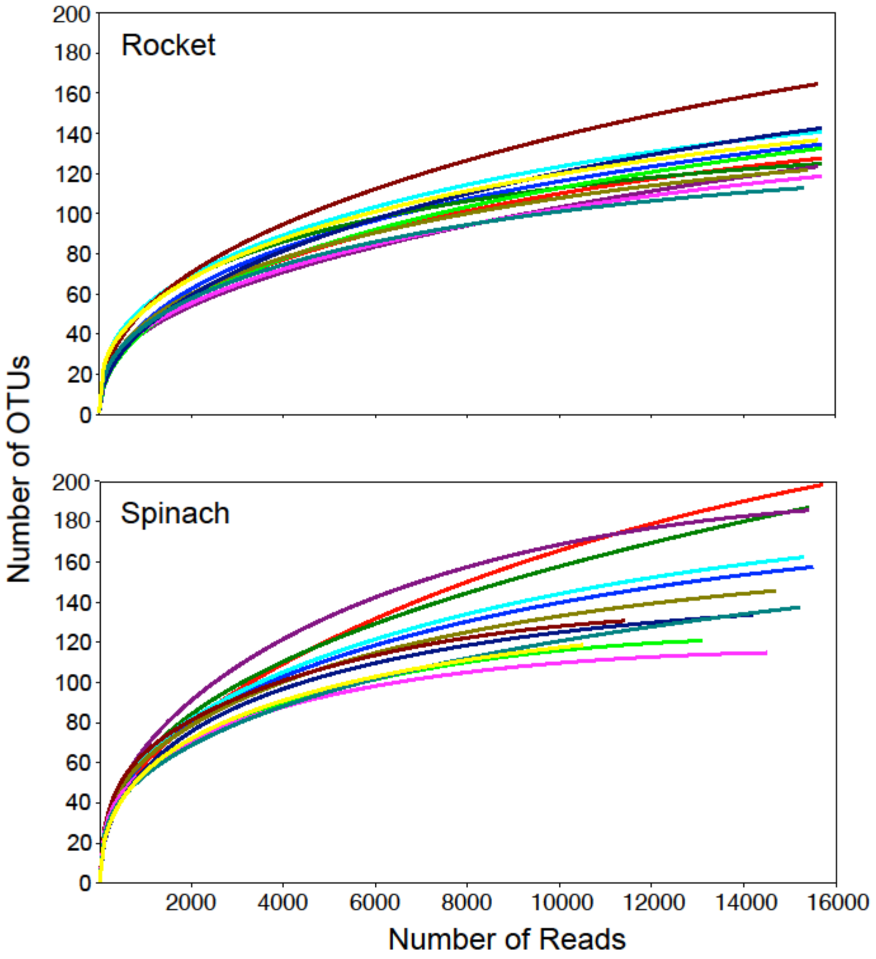

Supplementary Figure S1. Rarefaction curves representing the number of OTUs against the number of high-quality reads.

Supplementary Table S1: OTUs number, the richness estimator S_{chao1} and α-diversity measurements (Simpson, Shannon and Equitability) per sample.

Samples	Number of OTUs	S_{chao1}	Simpson	Shannon	Equitability
RN1	128	151.8	0.62	1.80	0.37
RN2	135	169.2	0.73	2.00	0.41
RN3	125	135.5	0.84	2.57	0.53
RN4	141	157	0.85	2.61	0.53
RW1	119	142.8	0.64	1.87	0.39
RW2	143	182.2	0.58	1.65	0.33
RW3	113	124	0.84	2.37	0.50
RW4	137	159.5	0.91	2.91	0.59
RV1	124	175.3	0.56	1.65	0.34
RV2	133	172.4	0.54	1.56	0.32
RV3	122	134.2	0.86	2.39	0.50
RV4	165	216.8	0.80	2.27	0.44
SN1	199	243.3	0.76	2.49	0.47
SN2	158	178.8	0.89	2.85	0.56
SN3	188	300.5	0.88	2.72	0.52
SN4	163	184.8	0.90	2.96	0.58
SW1	115	115.8	0.87	2.81	0.59
SW2	134	140.6	0.84	2.50	0.51
SW3	138	175.3	0.90	2.85	0.58
SW4	119	132	0.87	2.63	0.55
SV1	186	191.8	0.88	2.90	0.56
SV2	121	123	0.87	2.70	0.56
SV3	146	160.5	0.92	3.08	0.62
SV4	131	135.1	0.90	2.97	0.61
